# Medical Opinion and Movement

**Published:** 1910-08-13

**Authors:** 


### Medical Opinion and Movement.

### Oral Pulsatile Expiration.

The effect of the heart on the adjoining tissues often determines certain phenomena which have as a common characteristic a rhythmic nature coincident with the phases of the cardiac action. Many of these phenomena are only apparent in pathological conditions, and in consequence possess a diagnostic value. An interesting sign of this kind is recorded by Dr. Ernst Freund in the *Zeitschrift für klinische medizine*, to which he gives the term oral pulsatile expiration. The patient was a miner, aged thirty-four, with exaggerated hypertrophy of the left ventricle and a chronic infiltration of the upper lobe of the left lung. The patient suffered from increasing dyspnœa with accelerated pulse amounting to 132 per minute. On auscultation a number of phenomena were observed, due to the communication of the cardiac activity to the adjoining lung tissue—cardio-pulmonary bruits, systolic vesicular murmur, pulsatile respiration, etc. Cardiac râles could also be heard—that is to say, râles synchronous with the activity of the heart—and heard even during cessation of respiration. Furthermore, if the ear were placed to the open mouth a reinforcement of the respiratory murmur was heard during expiration, which was produced in jerks following the rhythm of the heart. This rhythmic respiratory shock could be quite clearly detected even when the patient was instructed to hold his breath towards the end of expiration. It produced the impression as if at each cardiac phase a certain amount of air was expelled with force from the mouth. As far as one could judge, the phenomenon coincided with cardiac systole.

### Radium for Post-operative Cicatrices.

An interesting series of cases is reported in the *Journal de Médecine de Paris* by Dr. S. Fabre, in which radium has been employed with a view to reducing hard and painful cicatrices and adhesions following abdominal operations chiefly of a gynæcological nature. One case was that of a woman who had undergone three operations for appendicular and pelvic abscesses. She had a hard cheloid and painful cicatrix with adhesions extending to the right ovary. The periods were irregular, scanty, and painful. Radium applications were made externally to the abdominal cicatrix and also within the vagina. After three months' treatment, the applications being made at intervals of two or three weeks, the cicatrix became quite supple, the cheloid disappeared, the menstrual periods became normal, and the general condition of the patient improved. In another case there was a median painful cicatrix with adhesions to the bladder following a hysteropexy. Similar treatment with radium was followed by the same beneficial results. In a third case there was a median vaginal cicatrix following hysterectomy for fibroid. The cicatrix was hard and very painful. Three applications of radium of five to six hours' duration were sufficient to remove the pain and also render the cicatrix much more supple. The duration of the vaginal application was six to twelve hours, the abdominal ten to twenty-four hours. In obstinate cases the time may be prolonged. According to the author, the radium emanations exercise a selective action upon the cicatricial tissue without any ill-effects upon the surrounding healthy tissues. Occasionally a slight redness may result from a prolonged application, but this disappears in the course of a few days.

### Urinary Infection and Pregnancy.

Owing to the frequency of infection of the urinary tract during pregnancy or following parturition, Dr. P. Alsberg has carried out a bacteriological examination in a large number of cases. In fifty-five cases of pregnancy he found the bacillus coli present in the vestibule in 13 per cent., and in the urethra in 5.5 per cent. Following parturition these proportions are much increased. In 103 cases immediately after confinement he found the bacillus coli present on the fourth day in 51 per cent. for the vestibule and 50 for the urethra. On the eighth day the proportion was diminished to 42 and 31 per cent. respectively. In cases of abortion the figures were similar—namely, 40 and 43 per cent. From these facts the author argues that the vaginal secretions during pregnancy are probably bactericidal, but that the lochia are more favourable to the development of the bacillus coli. Under these circumstances catheterism during the puerperium is not without danger, and cystitis is very liable to supervene. The author is of opinion that the bacillus coli is responsible in a larger proportion of cases of cystitis in this way, and although the infection is relatively innocuous in comparison with other pathogenic micro-organisms, the bacillus coli shows a remarkable aptitude for spreading up the urinary passages and multiplying. Dr. Alsberg, for example, has found the bacillus in the ureters five days after the appearance of a cystitis. Of twenty-nine cases of cystitis following confinement, about one-half were attributable to the bacillus coli.

### Duration of Rest after Confinement.

It is already well known that a few bold spirits both in the gynæcological, and also in the surgical world, contrary to the results of the years and years of experience of our predecessors, advocate getting a patient up within the first three or four days after a normal confinement or laparotomy. The rapid advance in surgical technique no doubt fully justifies a reconsideration of all and every detail of treatment hitherto pursued, but in obstetrics, apart from precautions against sepsis, the changes in the treatment of a normal confinement would not appear to be so radical as to render past experiences of no account in regard to precautions against complications and sequelæ. Be this as it may, the observations of Dr. Darcanne-Mouroux in the *Journal de*

*Médicine de Paris* are worthy of consideration, in view of these somewhat revolutionary ideas. In Brittany women of the peasant and working class consider it a question of duty and honour to be up and about on the second to the eighth day after a confinement. Dr. Darcanne-Mouroux has examined a hundred such women several times at varying intervals after a confinement, and the following are the results of these observations:—

| | Cases. |
|---|---|
| Normal condition of the sexual organs ... ... | 27 |
| Subinvolution of the uterus and consequent metritis ... ... ... ... ... ... ... | 22 |
| Prolapse of the second degree ... ... ... | 20 |
| Retroversion ... ... ... ... ... ... ... | 13 |
| Exaggerated antiversion ... ... ... ... | 6 |
| Cystocele ... ... ... ... ... ... ... | 6 |
| Inflammatory lesions of the appendages ... | 5 |
| Phlebitis ... ... ... ... ... ... ... | 1 |

Cystocele complicated the prolapse or retroversion in eleven cases. The author points out further that these abnormal conditions did not appear to follow more frequently in women who had returned to work early than in those who simply sat up. Nor did they appear due to multiparity, as the normal cases included women who had had seven, ten, thirteen, fourteen, and sixteen children. These observations would appear, therefore, to offer a direct negative to this new "profession of faith." They show a proportion of 73 per cent. of lesions following normal confinements, and the most frequent is uterine subinvolution, which the new method of treatment is supposed especially to avoid.

### Injections for Gonorrhœa.

Although injections of hydrogen peroxide were advocated by Dr. Lowe in the treatment of gonorrhœa some years ago, the method does not appear to have received general recognition. According to Dr. A. Skoulsky, however, most satisfactory results are obtained by these urethral injections, and he ascribes their success to the power of diffusion as well as the bactericidal properties of hydrogen peroxide. They are at the same time much less irritating to the mucous membrane than the silver preparations usually employed. Several cases of acute and chronic gonorrhœa are reported in detail in which the treatment was successfully employed. In one case the patient had suffered from a chronic posterior urethritis for three months, and the usual injections of silver nitrate, permanganate of potash, etc., had failed to cure. The author commenced with injections of 20 c.c. of hydrogen peroxide diluted to half strength twice a day, the fluid being retained for three minutes. The amount was gradually increased to 50 c.c., and at the same time the strength was gradually increased till the hydrogen peroxide was injected undiluted. At the end of a fortnight the patient was cured, and bacteriological examination of the urine was negative and remained so. Another case is recorded in which a chronic posterior urethritis was of three years' duration, and similar treatment for a fortnight also resulted in a cure, with complete disappearance of the gonococci in the urine. In acute cases the author does not advise the injections till the second or third week.

### Spinal Injections for the Insane.

A remarkable case is recorded by Dr. J. Roubinovitch in the *Journal de Médicine de Paris*, in which a condition of alcoholic insanity showed undoubted improvement under a treatment of spinal injections of cerebro-spinal fluid from a horse. The injections were followed by an aseptic inflammatory meningeal reaction, and the improvement which set in appeared to be largely attributable to this fact. The patient was a man aged forty-three, addicted to alcoholic excess, and admitted to the Bicêtre Hospital suffering from melancholic delirium, with illusions, active tendency to suicide, and ideas of persecution. The prostration was extreme with anorexia, trembling of the hands, subnormal temperature, scanty urine, restless sleep, and auditory hallucinations. In spite of treatment the mental and physical depression persisted, and the author decided to try the method of treatment he has already successfully carried out in several cases of asthenia. Five c.c. of cerebro-spinal fluid were first evacuated, and an equal quantity of cerebro-spinal fluid from a horse was then injected. The evacuated fluid was found on examination to be quite normal. This procedure was rapidly followed by an acute meningeal reaction. The temperature rose to 38° C. and then to 40°.7 C. a few hours after injection, accompanied by vomiting and an outbreak of herpes labialis. The temperature remained above normal till the fourth day. The patient remained prostrate and silent, but less troubled and anxious. Seven days after the injection he said he felt better, and wished to get up. Examination of the cerebro-spinal fluid showed the presence of a lymphocytosis. The bodily symptoms steadily improved, and he gained in weight. This improvement, both mental and physical, continued, and at the end of three weeks he was about helping the nurses and apparently in a normal condition. He had gained 7 kilos. in weight. Questioned, however, in regard to his previous illusions, he showed a firm belief in their reality. The author thinks that these impressions will also gradually disappear. In any case the improvement resulting from the injection and subsequent meningeal reaction was remarkable, and encouraging for further experimental treatment on the same lines.

### Local Anæsthesia of the Pudendal Nerve.

Local anæsthesia by injections of cocaine solution around the trunk of the pudendal nerve was first introduced by Dr. W. B. Müller in 1908. It has since been employed for a number of operations in the area supplied by that nerve, and the technique has been improved and defined quite recently by Dr. Ilmer, of Klagenfurt, and Professor Sellheim, of Tubingen. The pudendal nerve, it will be remembered, leaves the pelvis by the great sacro-sciatic foramen, and reaching the surface by curving around the tuber ischii, divides into the inferior hæmorrhoidal, superficial perineal and dorsal nerve of the clitoris or penis. The injection is made internal and somewhat posterior to the tuber ischii, the needle being directed towards the sacro-sciatic notch. In very obese subjects the site is found by measuring three fingers' breadth external to the anus; in others the tuber ischii forms the guide.

Ilmer recommends the use of a syringe of 10 cubic centimetres capacity, and injects 5 per cent. cocaine in normal saline solution. The needle should be 5 c.m. long. The injection is made at the point indicated, and to the right and left of it on each side of the body. Anæsthesia is complete in fifteen to twenty minutes. This form of anæsthesia is applicable for peri-neorrhaphies and hæmorrhoid operations. Its use is suggested also in confinements to diminish the pains, especially in forceps cases.

### Pulmonary Tuberculosis.

The occurrence of inequality of the pupils in cases of unilateral pulmonary tuberculosis has been recognised for a long time, but this symptom has appeared so rare that it has not been regarded as a useful sign in point of diagnosis. According to Dr. Fodor, however, this phenomenon is much more common than has been supposed, but its recognition requires certain special precautions. In broad daylight the inequality is not in evidence on account of the pupils being more or less contracted. If, however, the comparison between the two pupils is made in a dim light, it will be found that the pupil corresponding to the affected side dilates more rapidly and to a greater extent, so that at the end of the reaction it is larger than the other. If now a light is brought upon the eyes this same pupil contracts more feebly and more slowly than its fellow. This difference appears to be quite independent of the intensity or extent of the pulmonary lesion. The author has obtained this sign in cases in which the objective signs in the lungs were as yet absent, the hereditary antecedents and general condition of the patient alone raising the suspicion of the presence of the disease, which was only confirmed later by the development of pulmonary signs. This inequality of pupils may be determined not only by unilateral pulmonary tuberculosis, but also by any other affection involving only one side of the chest—tumour, aneurism, pleurisy, etc.—and capable of setting up an irritation of the sympathetic nerve. But in any case this phenomenon should prove a useful diagnostic sign in cases of early tubercular infection of one lung, in which the ordinary pulmonary signs are wanting.

### The Specificity of Cancer.

Dr. Alexander T. Brand, in the course of an address delivered to the East Yorkshire Division of the British Medical Association, makes a strong plea for the recognition of cancer as a specific disease. He rapidly reviews the reasons for considering cancer as being such and quotes authorities in support of his contentions. He believes that in spite of surgical treatment being in many cases unsatisfactory, it alone, in the present state of our knowledge, holds out any prospect of a cure. Should the future reveal the causal agent of the disease to be a specific organism, success may follow the exhibition of a cancer vaccine prepared after the method of Wright, or of an anti-cancerous serum. In the meantime everything that tends towards the prophylaxis of the disease should be tried. The cancerous cadaver should be cremated. All contaminated dressings from cancerous patients should be destroyed. Intimate personal contact with the diseased should be avoided, and all food and drinking water sterilised. Raw vegetables and uncooked fruits should be abstained from, especially those which lie on or in the ground. Finally every case of cancer should be notified.

### Oleic Acid the Cause of Eclampsia?

The question as to whether oleic acid derived from the placenta is the cause of the convulsions in puerperal eclampsia is discussed by O. Polano in the *Zeit. f. Geburts. u. Gyn.* The acid is found in the villi, the membranes and the cord of normal puerperal patients, and sodium oleate in the blood. It is obvious that if the acid has anything to do with the production of the fits it must be present in the tissues mentioned in more than physiological proportions in eclamptic patients. Moreover, it must cause anæmia by the poisonous action of sodium oleate on the blood-cells, and this effect must be sudden. It could only result from the direct action of the salt on the central nervous system, but it is known that sodium oleate does not produce such an effect. The author has made a careful examination of two normal placentæ and of two in which their owners died from severe eclampsia. He has also investigated the blood obtained by venesection from a case of eclampsia, and that of a nongravid case of carcinoma, as well as the urine obtained immediately, and at an interval of three to five days, after an eclamptic fit. He finds oleic acid present in equal proportions in normal and eclamptic cases. Eclamptic blood contains no more sodium oleate than normal blood. There is no difference in the urine obtained immediately after and that collected a few days after a fit. Blood cells are increased in cases of eclampsia. He therefore concludes that the balance of evidence is against oleic acid having any effect in the causation of the condition.

### Opiates in Infancy.

The much-discussed question of the dangers of giving opiates to young children is once more dealt with in the *Annales de Médicine et de Chirurgie infantile* by Dr. Lust. This observer says that there is no contraindication to their use in infants, and that they are of the greatest value in spasmodic conditions. He declares, however, that it is not safe to prescribe opium preparations which contain all the alkaloids of the drug. Morphine alone should be used. The tolerance of infants for this alkaloid is greater than that of the adult if the dose be calculated from the weight of the patient and not, as is usually the case, from its age. This alkaloid is stable and its dose can be carefully regulated. If given by the mouth the dose should be calculated at one milligram for each kilogram of weight for twenty-four hours. This dose should be halved if given subcutaneously. It can be increased without danger as there is no accumulation in the system.

